# Venoms and Extracellular Vesicles: A New Frontier in Venom Biology

**DOI:** 10.3390/toxins17010036

**Published:** 2025-01-14

**Authors:** Auwal A. Bala, Naoual Oukkache, Elda E. Sanchez, Montamas Suntravat, Jacob A. Galan

**Affiliations:** 1 Department of Human Genetics, School of Medicine, University of Texas Rio Grande Valley, Brownsville, TX 78520, USA; auwal.bala@utrgv.edu; 2 Laboratory of Venoms and Toxins, Pasteur Institute of Morocco, Casablanca 20360, Morocco; naoual.oukkache@pasteur.ma; 3 Department of Chemistry and National Natural Toxins Research Center, Texas A&M University-Kingsville, Kingsville, TX 78363, USA; elda.sanchez@tamuk.edu (E.E.S.); montamas.suntravat@tamuk.edu (M.S.)

**Keywords:** exosomes, extracellular vesicles, EV-omics, envenoming, acute injury, biomarkers, toxins, venom

## Abstract

Extracellular vesicles (EVs) are nanoparticle-sized vesicles secreted by nearly all cell types under normal physiological conditions. In toxicological research, EVs have emerged as a crucial link between public health and multi-omics approaches, offering insights into cellular responses to disease-causing injury agents such as environmental and biological toxins, contaminants, and drugs. Notably, EVs present a unique opportunity to deepen our understanding of the pathophysiology of envenomation by natural toxins. Recent advancements in isolating and purifying EV cargo, mass spectrometry techniques, and bioinformatics have positioned EVs as potential biomarkers that could elucidate biological signaling pathways and provide valuable information on the relationship between venomous toxins, their mechanisms of action, and the effectiveness of antivenoms. Additionally, EVs hold promise as proxies for various aspects of envenomation, including the toxin dosage, biological characterization, injury progression, and prognosis during therapeutic interventions. These aspects can be explored through multi-omics technology applied to EV contents from the plasma, saliva, or urine samples of envenomated individuals, offering a comprehensive integrative approach to understanding and managing envenomation cases.

## 1. Introduction

Animal venoms originating from a wide variety of species, such as snakes, scorpions, and spiders, are a complex mixture of biologically active molecules that venomous species use to capture and digest their prey. These active compounds comprise non-enzymatic and enzymatic peptides/proteins, as well as small molecules. During envenomation, these toxins target multiple cellular systems, affecting inflammatory responses, cellular apoptosis, neurophysiological and neuromuscular functions, and hemostasis, ultimately leading to the paralysis or death of the prey [1,2].

Traditionally studied for their neurotoxic, cytotoxic, and myotoxic effects, these venoms are a rich source for medical biotechnology with numerous therapeutic applications, ranging from analgesics to cancer treatments [3]. However, a previously overlooked component of venoms is now attracting increasing attention from researchers: extracellular vesicles (EVs). These membrane-bound microparticles, capable of transporting biomolecules throughout the organism, offer a new perspective on the mechanisms of venom action.

EVs, which include exosomes and microvesicles, are structures released by cells and present in many bodily fluids. They play a crucial role in intercellular communication, transferring their cargo proteins, lipids, RNA, and other biomolecules from one cell to another. Recent studies have revealed that venoms might also contain EVs, a discovery that redefines our understanding of how venom toxins are distributed and how they exert their effects [4,5]. Although the potential of EVs as carriers of information or toxins within targeted organisms is still in its early stages, it represents a promising new frontier.

This discovery adds a new dimension to our understanding of venoms: (1) a deeper understanding of envenomation: EVs may help transport toxins to target cells, enhancing the venom potency and explaining variations in symptoms; (2) new treatment strategies: targeting EVs could revolutionize antivenom design by disrupting toxin dissemination, offering a fresh therapeutic approach; and pharmacological potential: venom-derived EVs might be used in drug delivery systems, capitalizing on their bioactive peptide content for precise therapeutic targeting. This emerging field could lead to significant advancements in venom research and medical applications.

Despite over 600 venomous species having been explored, only 15–20% have been thoroughly characterized using proteomics and transcriptomics technologies to understand their venom composition, toxicological relationships, and mechanisms of action. This gap in knowledge highlights the need for more comprehensive proteomics, transcriptomic, and machine learning/artificial intelligence analyses. Recent insights into EVs have added another layer of complexity to these already fascinating and toxic mixtures.

This review aims to explore EVs and exosomes, focusing on their emerging role as key players in venoms and venom-induced responses. We summarize the latest discoveries on venom EVs from snakes, wasps, spiders, scorpions, ticks, and cone snail venom and delve into the intricate features, isolation methods, and transmission of EVs/exosomes in the context of envenomation. This review addresses how toxic molecules alter exosome formation and composition and examines the impact of venom/toxins on the cargo carried by EVs/exosomes. Furthermore, we highlight the potential role of EVs and exosomes in transmitting acute and systemic pathophysiological effects, inflammation, tissue damage, and disease progression resulting from venom-related injuries. By exploring these aspects, we aim to shed light on the significant implications of EVs and exosomes in venom-induced pathologies and their potential impact on human health.

### 1.1. Animal Venom

**Figure 1:**
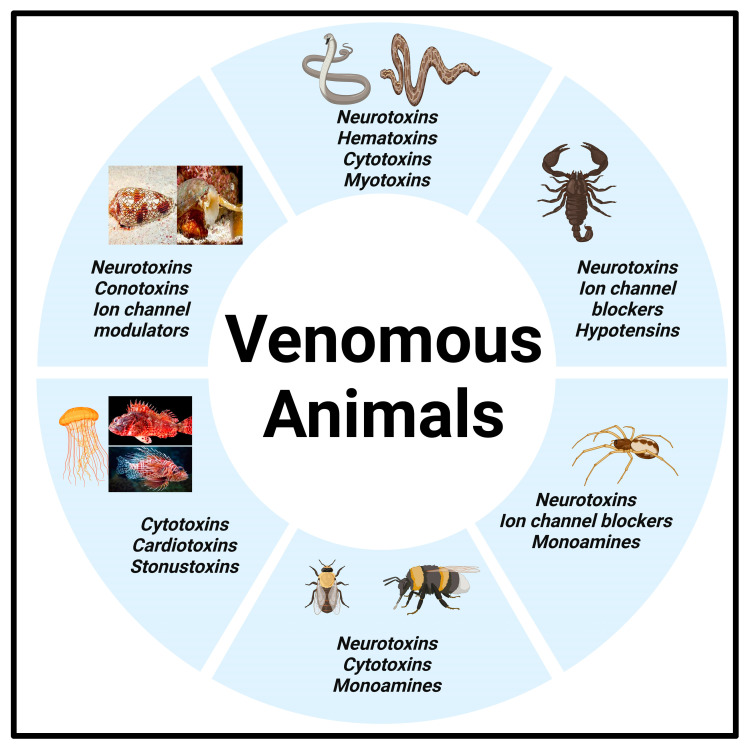
Venom components of some venomous animals.

Venom is a mixture of bioactive molecules used by a diverse group of animals that aids in the capture and digestion of their prey. Venoms are usually mixtures of many different components, including salts, amino acids, neurotransmitters, and other organic compounds, but primarily contain peptides and proteins with toxic activities called toxins [6,7]. These toxin mixtures can range from simple to complex and are delivered through bites, stings, and scratches. Venoms are found in both marine and terrestrial animals, vertebrates and invertebrates (Figure 1). These venomous mixtures contain notable toxins such as necrotoxins and cytotoxins affecting cellular function and apoptosis; neurotoxins affecting the nervous system and causing paralysis; myotoxins causing muscle damage; and hemotoxins affecting hemostasis, causing hemorrhages and blood clots [6]. Venoms evolve to their environment, which includes the geographical and temperature/climate terrain, having genetic selection properties for their toxins and evolving to become maximally efficient in killing or at least disabling their specific prey items. For example, venom toxins target particular receptors or lethal components like blood clotting factors, platelets, and nicotinic receptors that cause pathophysiological injury, such as perturbations in coagulation and paralysis within the prey [7,8]. The resulting predator–prey interactions utilizing their venom become specialized to an animal’s standard diet.

Venoms can be found in invertebrates, including arthropods, such as spiders [9], scorpions [10], and wasps [11], in the phylum cnidaria, including dangerous box jellyfish, siphonophores, and sea anemones [12,13], in echinoderms such as sea urchins, and in mollusks like cone snails and octopuses [14,15]. They can also be found in vertebrates such as fish, amphibians, reptiles, and mammals [6,16]. The milieu of toxins is expressed, secreted, and stored in a highly evolved salivary gland and sac, and due to the diversity and range of venomous species, it presents a complex genetic architecture unique to the species.

### 1.2. Medically Relevant Venomous Species: Snakes, Scorpions, Spiders, and Cone Snails

Tropical and rural regions across the world face health issues related to animal species that possess the ability to inject venom that causes morbidity and mortality and sometimes leads to therapeutic discoveries. These species include sea snakes, cnidarians, poisonous fish, spiders, snakes, and scorpions [17]. Venom’s complex mixture of extremely potent biologically active substances changes between different species, subspecies, or geographic variations that can produce a wide range of venom compositions, clinical indications, and symptoms. However, venom comprises many compounds with unique biological properties that have applications in biomedical sciences and pharmacology, including the discovery of alpha and beta neurotoxins that significantly affect the nicotinic acetylcholine receptors (nAChRs) [18,19]. In certain circumstances, the toxins can interfere with neurotransmitters like gamma-aminobutyric acid (GABA), noradrenaline, adrenaline, and dopamine, leading to the distortion of neuromuscular transmission at the neuromuscular junction [20]. These interactions provide valuable insights into physiological mechanisms and contribute to the understanding of various pathological processes [19,20]. Additionally, the study of venom compositions across diverse species has revealed critical mechanisms of envenomation, which play a significant role in the development of therapeutic drugs [6]. Despite this progress, a thorough investigation into the molecular mechanisms underlying the action of animal venoms and their associated toxins is essential for medical advancements. A comprehensive understanding of venom composition is vital for enhancing the efficacy of envenomation management strategies.

#### 1.2.1. Snakes

Venomous snakes possess fangs that are connected to venom glands responsible for producing venom, which could be the solenoglyphous fangs found in vipers, which are long, hollow, and hinged, folding against the roof of the mouth when not in use. Elapids, such as cobras, have proteroglyphous fangs that are short, fixed, and positioned at the front of the maxilla. Some rear-fanged snakes, like colubrids, possess opisthoglyphous fangs, grooved and located farther back in the mouth, delivering venom more gradually. Unlike solenoglyphous or proteroglyphous species, they typically lack a dedicated venom gland directly connected to these fangs. Because the snake venom composition varies at the species, subspecies, and even geographic variation levels, it can cause a wide range of clinical symptoms after envenomation. Most snakebite envenomings worldwide are caused by venomous snakes, particularly in rural, tropical, developing nations in Asia and Africa. Tissue damage is the primary cause of viperid snakebite morbidity, which can result in lasting disabilities, such as contractures, hypertrophic scars, persistent ulceration, chronic renal disease, eye damage, crippling maladies, and a permanent loss of muscular tissue [2]. These venoms often comprise hematotoxic components, such as metalloproteases that disrupt clotting mechanisms, alongside calcicludine and protamine, which inhibit sodium (Na^+^) or calcium (Ca^2+^) ion channels [1,2,21]. On the other hand, envenomation by neurotoxic snakes, primarily elapids, can induce neurotoxicity, cytotoxicity, and cardiotoxicity, leading to death from neuromuscular paralysis and life-threatening respiratory dysfunction [22]. Neurotoxic effects may arise from toxins that interfere with synaptic transmission. For instance, three-finger toxins (3FTxs) and phospholipase A_2_ (PLA_2_) act as antagonists of cholinergic receptors, thereby blocking acetylcholine receptor activity [23].

#### 1.2.2. Scorpions

Scorpion envenomation is prevalent in tropical and subtropical regions, representing a significant public health concern due to the potential for severe clinical symptoms and fatal outcomes, including death [10,24]. Scorpion stings are among the leading cause of envenomation following snakebites [10,25]. Due to the morbidity linked to their venoms, scorpion genera such as Centruroides (found in North and Central America, including Colombia), Tityus (South America), Androctonus, Leiurus, Buthus (North Africa and the Middle East), Parabuthus (South Africa), and Mesobuthus (India) generally garner the most attention in various countries and the pertinent related literature [24,26]. Scorpion envenomation is recognized as a major public health problem in the Middle East and North Africa [24,27]. The primary cause of death from scorpion envenomation includes cardiogenic shock and pulmonary edema, which is caused by the release of substances that trigger sympathomimetic changes [10].

Scorpion venom exhibits a broad range of biological activities. The central and peripheral nervous systems’ architecture is impacted by the toxins found in scorpion venom, which include neurotoxins (alpha and beta toxins, phospholipases), enzymes (acetylcholinesterase), hemotoxins (metalloproteases), and ion channel blockers [28]. These effects can result in paralysis, convulsions, brain inflammation, and hemorrhagic and ischemic strokes [29]. Peptides are among the complex combinations of chemicals found in scorpion venom. These toxins modulate the Na^+^, Ca^2+^, K^+^, and Cl⁻ channels [30]. Other peptides found in scorpion venom include hypotensins, metal-chelating peptides, neurotoxins, and antibiotics [31].

#### 1.2.3. Spiders

Equipped with complex poison, spiders are among the most adept predators in nature. While spider bites can cause discomfort and localized symptoms, they seldom result in envenoming that poses a serious risk to life. The primary local effects associated with bites from Loxosceles species include dermonecrosis and subsequent infections [32]. The main purpose of spider venom is to paralyze prey by blocking ion channels with neurotoxins. These venoms mostly consist of peptides such as neurotoxins, ion channel blockers, and monoamine neurotransmitters, which have a variety of biological actions [9,33]. Due to the development of specialized venom glands, spiders and scorpions have become the most notable venom producers in the arachnid family. Except for several species of Mesothelae, Uloboridae, and Symphytognathidae, most spiders have chelicerae that include venom glands [34]. Black widow spiders (a *Latrodectus* species) are among the most medically significant spider species due to their strong myotoxic and neurotoxic effects. Spider venoms are sophisticated biological substances which mostly target particular ion channels through the action of neurotoxins [35]. Composed of intricate mixtures, these venoms include a variety of components such as proteins, polypeptides, neurotoxins, nucleic acids, free amino acids, inorganic salts, monoamines, and low-molecular-weight organic components [9,36]. This complex composition underscores the diverse mechanisms by which spider venoms exert their effects.

#### 1.2.4. Marine Snails

The *Conus* genus, part of the Conidae family, is a group of predatory gastropod mollusks, some of which pose significant dangers to humans and other animals. Envenomation is most likely to occur during handling by divers and shell collectors, with envenomation primarily affecting the palms and fingers [15,37]. *Conus* venom contains complex bioactive toxins, including neurotoxins (latrotoxin and atracotoxins) and conotoxins (ω-conotoxin and α-conotoxin) that cause paralysis by inhibiting the neuromuscular pathway [14,38]. The combinations of peptides that form Conus venom vary between species, containing thousands of distinct bioactive toxins. These toxins have various neuromuscular effects via glutamate, adrenergic, serotonergic, and cholinergic pathways. Some conotoxins target Na^+^, K^+^, and Ca^2+^ ion channels [15]. A study reported the transcriptomes from the carcass, salivary glands, and proximal and distal venom ducts of the northeastern Atlantic species *Raphitoma purpurea*, revealing evidence of a vermivorous diet for the genus [39]. The transcriptomic analysis identified over a hundred potential venom components, including sixty-nine neurotoxins [39]. The complexity of this toxin and variations in target pathways have made it difficult to develop an effective antivenom [15,40].

#### 1.2.5. Bees/Wasps

Honeybees are distributed worldwide and play a crucial role in pollination in both natural ecosystems and agricultural settings. As most bee species are generally docile, most interactions with humans and bees are unproblematic despite their ability to inject sophisticated venom into their prey as a defensive tactic. Nonetheless, bee stings have increased since the unintentional release of Africanized bees into Brazil in 1956 and their subsequent spread [41]. Most bee stings do not cause death but frequently produce local, minor allergic reactions in people, but a single bee bite can result in a lethal anaphylactic reaction [42]. Normally, an anaphylactic reaction is the cause of mortality; however, when a child gets several stings (more than 30), the direct toxicity of the venom can lead to death [43]. Bee venom is a complex collection of chemicals, including proteins, peptides, amino acids, phospholipids, sugars, biogenic amines, volatile compounds, pheromones, and water (>80%). The composition of bee venom has already been characterized using omics methods and venom fractionation [41,44,45]. Wasps, members of the Hymenoptera order, can be found in various regions worldwide, including Brazil, Thailand, Japan, Korea, and Argentina. They may live solitary and gregarious lifestyles. Chemically, wasp venom contains a diverse range of enzymes, proteins, peptides, volatile compounds, and bioactive substances, including PLA_2_, antigen 5, mastoparan, and decoralin. These bioactive ingredients can cause cytotoxic, genotoxic, mutagenic, and hematotoxic effects [11].

#### 1.2.6. Venomous Fishes

Fish account for more than half of all poisonous vertebrates, putting divers at risk all around the world. The most venomous fish belongs to the family Scorpaenidae. Lionfish, scorpionfish, and stonefish are all members of this family [46,47,48]. Venomous fish stings can be agonizingly painful. Mechanical injury damages tissue, and the local action of the injected venom causes more harm. In rare cases, severely penetrating stings can damage massive blood vessels and nerves. Some types of venomous fish can cause systemic envenoming [17]. The Scorpaenidae family of fish causes headaches, weakness, diaphoresis, nausea, vomiting, abdominal discomfort, hypotension, chest pain, cardiac arrhythmias, myocardial ischemia, syncope, and even pulmonary edema [13]. They have heat-labile, non-dialyzable venoms with varying potency but a strikingly similar composition. The toxins are made up of high-molecular-weight (50 to 800 kDa) proteins called hyaluronidase, pain-producing factors, capillary permeability factors, and species-specific toxic factors [46,47,48,49]. Jellyfish can envenomate people; however, most are harmless to humans because they lack nematocyst shafts of a sufficient length to allow the thread to deposit toxins deep enough into the epidermis or may create toxins that cause no major harm to humans [12]. Toxic cnidarians include vertebrate feeders or larger jellyfish capable of releasing significant amounts of toxins [12].

#### 1.2.7. Ticks

Ticks are rarely thought of as venomous animals, even though their saliva contains many protein families found in venom taxa and many Ixodida species can cause paralysis and other toxicoses [50]. The functions of several families of tick salivary proteins and those found in the venoms of snakes, bees, scorpions, platypuses, and spiders are comparable [50]. Saliva from a number of tick species, including Dermacentor andersoni, Argas walkerae, and Ixodes holocyclus, has been found to have poisons that paralyze people. By disrupting the production and lowering the release of acetylcholine at the neuromuscular junction, these species have been shown to cause paralysis [51,52,53].

### 1.3. The Diversity of Venoms and Their Clinical Relevance

There are thousands of venomous animals, including snakes, scorpions, spiders, bees, cone snails, and venomous fish, many of which are medically significant due to accidental interactions with humans. The composition and biological effects of the venom depend on the family, species, age, and geographical location. While certain toxins are shared across species with similar biological activity, the level of toxin abundance can vary significantly between animals, species, and even intraspecies (Table 1).

## 2. Extracellular Vesicles (EVs)

EVs are nanoscale vesicles secreted by almost all types of cells, including healthy and diseased cells; they are heterogeneous but with similar characteristics [56,57]. The recent use of EVs and their critical cellular functions has presented them as an intriguing source for novel biomarker discovery and disease diagnosis [56,58]. EVs play crucial roles in intercellular communication, modulating various biological processes, including apoptosis, immune responses, and tissue homeostasis [58]. These nanoscale membrane-bound particles have been successfully isolated from diverse biological fluids, including blood, urine, saliva, breast milk, and cerebrospinal fluid [59,60].

The unique molecular cargo of EVs, including proteins, nucleic acids, and lipids, reflects the physiological or pathological state of their cells of origin. This characteristic has positioned EVs as promising diagnostic and prognostic tools for various diseases [61]. In oncology, EV-associated biomarkers have shown potential for early cancer detection, monitoring the treatment response, and predicting metastasis [62,63]. In the context of neurodegenerative diseases, EVs have emerged as potential biomarkers for conditions such as Alzheimer’s and Parkinson’s disease, offering insights into disease progression and potential therapeutic targets [64,65]. Furthermore, in cardiovascular medicine, EV-based biomarkers have demonstrated utility in predicting cardiovascular events and assessing myocardial injury [66,67]. The rapidly evolving field of EV research continues to uncover new applications and refine existing methodologies, promising to revolutionize disease diagnosis, prognosis, and personalized medicine approaches across various medical disciplines.

The role of EVs from disease-causing agents like pathogenic microorganisms has been well-documented and emerged as an important link between toxicological research and public health [68,69,70]. More recently, it has been shown that EVs respond to exposure to environmental toxins, contaminants, and drugs, highlighting their potential in toxicology [71,72]. EVs have recently been collected, separated, and analyzed in both clinical and epidemiological toxicology research, providing important information on pathophysiological communication in the exposomes of different toxins from vertebrate animals, fungi, and bacteria [4,68,73].

### 2.1. Types, Size, Structure, and Biogenesis of EVs

EVs constitute a variety of membrane vesicles that are released from the cell. They can be classified into three classes: apoptotic bodies, microvesicles (MVs), and exosomes, with different biological presentations and functions [74,75]. The cargo content comprises biologically essential proteins, lipids, metabolites, and nucleic acids, making them an important target in biological research [76,77]. Apoptotic bodies are the largest EVs, 1000–5000 nm in size. They are released from apoptotic blebbing cells (external forms of the cells) that are undergoing cell death. MVs range in size from 100 to 1000 nm and are generated by external budding from the plasma membrane. Exosomes make up the smallest EVs, having the smallest size of 30–100 nm, and are from the endosomal system [58,76]. EVs are released into the extracellular space, deliver information to other cells, and carry essential cargoes in communication between cells [61].

EVs have emerged as valuable tools in the pathological identification of rare diseases caused through the cellular and molecular pathway in cell communication, including homeostasis, and other diseases such as cancer and neurodegenerative disorders [61,78,79,80]. EVs have also been reported to play direct roles as pathogenic EVs, especially in neurodegenerative disorders, cancer, and even microbial infections [79]. The roles of EVs in homeostasis cannot be over-emphasized since EVs secreted from cells can travel through the circulatory system to deliver information to neighboring cells or cells in different locations [71].

### 2.2. Venom EVs: Types, Isolation, Characterization, and Function

#### 2.2.1. Snake Venom Extracellular Vesicles (SVEVs)

Snake Venom Extracellular Vesicles (SVEVs) are membrane-bound vesicles released from the venom glands. Though SVEVs were first observed in 1973 in snakes [81], recent studies have also detected and characterized these vesicles in the venom of bees, wasps, spiders, and ticks. In order to enrich EVs, they must be isolated from venom, and many methods are currently being used, such as differential and ultracentrifugation, size exclusion chromatography (SEC), extracellular vesicle total recovery and purification (EVTRAP), polymers, and antibodies, each with advantages and disadvantages.

##### Types and Isolation of SVEVs

The fact that EVs are generally nanoparticle-sized vesicles leads to the employment of different methods in isolating and purifying them from various sources, targeting sizes, types, and biological activity. The isolation of EVs from proteins such as snake venom requires special consideration, depending on the target EVs and the procedures after isolation [82]. Different methods have been employed to isolate venom EVs, especially from snakes [83]. An earlier study isolated membrane vesicles from *Crotalus durissus terrificus* membrane vesicles through the ultracentrifugation of cell-free venom, finding similarities in the size and structure to MVs observed in the glands. The venom was centrifuged at 5000 g for 15 min at 10 °C, followed by supernatant centrifugation at 240,000 g for 1.5 h at 10 °C to isolate the EV pellet, which revealed particles on the cytoplasmic leaflet (P-face) of these vesicles, suggesting that they carry transmembrane proteins [84]. Similarly, exosome-like vesicles (30–100 nm) from the venom of the pitviper *Gloydius blomhoffii blomhoffii* were isolated using SEC. They used fresh *G. b. blomhoffii* venom, which was centrifuged at 15,000 g for 5 min after being combined with 1000 μL of a buffer (20 mM Tris-HCl in 150 mM NaCl), followed by addition to a 1.50 cm Sephacryl S-300 sec column with the same buffer as pre-equilibration. The void fractions collected from the Sephacryl S-300 were concentrated using a centrifugal filtering system with a 100 kDa exclusion limit, and the analysis revealed that these fractions contained vesicles resembling exosomes [85,86]. Souza-Imberg et al. (2017) separated membrane vesicles from the venom of *C. d. terrificus followed* the centrifugation of the supernatant at 200,000 g [87]. Carregari et al. [5], used a size exclusion column (Superdex G75) to analyze *Agkistrodon contortrix contortrix*, *Crotalus viridis*, *C. cerberus oreganus*, and *C. atrox* venom EVs. The mixtures were centrifuged for 5 min at 9000 rpm and added to the column, and EVs were eluted in the void or early fractions. They further validated the presence of EVs using dynamic light scattering (DLS) and transmission electronic microscopy (TEM), revealing two populations of vesicles with diameters of 100 and 500 nm [5].

Recently, EVTRAP was used in isolating EVs from the venoms of *C. atrox*, *C. o. helleri*, and *C. s. scutulatus*. The lyophilized venom was centrifuged at 10,000 rpm for 10 min to remove cellular debris. EVTRAP beads were then added to the venom samples at a 1:100 *v*/*v* ratio, and the samples were incubated by shaking them or performing end-over-end rotation for 1 h, eventually eluting them with triethylamine [4,88]. Additionally, the EVTRAP method was used to isolate EVs from mouse plasma after injection with crude *C. s. scutulatus* venom, resulting in the isolation of a novel CRiSP toxin [88]. In a more recent study, differential ultracentrifugation (DU) was used to isolate EVs from the venom of *Bothrops jararaca*. Differential centrifugation at 20,000 g and 100,000 g allowed two size fractions to be characterized as P20K and P100K, respectively. They found the P20K fraction had vesicles of a particle size ranging from 30 nm to 1000 nm, and the P100K fraction had vesicles ranging between 30 and 130 nm in diameter [83]. DU is robust and results in protein aggregates, but additional clean-up steps are needed for the isolated EVs [83].

##### Characterization of SVEVs

EVs have recently been studied to understand their role, especially in the envenoming process. The release of intact membrane-bound vesicles has been observed in *C. viridis oreganus* [89]. The *C. d. terrificus* SVEVs were found to be MVs containing cytoplasmic proteins and proteins from the plasma membrane, endoplasmic reticulum, and Golgi membrane. The release of MVs may be a mechanism to control the size of the cell membrane of the secretory cells after intense exocytosis. These MVs’ content may have a role in envenoming [87]. Using liquid chromatography–mass spectrometric (LC-MS) methods on snake venom has allowed for identifying certain EVs related to snake venom, as reported in the venom of *Naja naja* and *Naja oxiana* [90]. Additionally, EV-associated proteins in the venoms from *C. atrox* and *C. o helleri* were purified using EVTRAP combined with mass spectrometry for the proteomic identification and quantification of SVEVs and plasma biomarkers. These vesicles were found to be enriched with proteins such as 5′-nucleosidase, LAAO, and other proteins. The analysis revealed upregulated responses associated with cytochrome P450, lipid metabolism, acute phase inflammation, and immune and heat shock responses, while downregulated proteins were linked with mitochondrial electron transport, NADH, TCA, the cortical cytoskeleton, reticulum stress, and oxidative reduction [4].

##### Toxic Function of SVEVs

Although EVs are well known for their role in cell–cell communication, they have also been found to play significant biological functions in snake venom. They exert effects similar to known venom proteins, including cytotoxicity, hematoxicity, hepatotoxicity, and paralysis akin to neurotoxicity. This is not surprising since the mass spectrometric proteomic analysis of EVs from *Agkistrodon contortrix contortrix*, *Crotalus atrox*, *Crotalus viridis* and *Crotalus cerberus oreganus*, and *Crotalus oreganus helleri* revealed eight venom protein families, including serine proteinases, PLA_2_s, LAAOs, 5′nucleotidase, CTLs, CRiSPs, disintegrins, and SVMPs with fibrinogenolytic effects [4,5]. A recent study used DU to purify and describe EVs from *Bothrops jararaca* venom and found that SVEVs may be involved not only in venom production and processing but also in host immune modulation and the long-term effects of envenoming. An LC-MS analysis revealed that the most abundant protein family is the 5′-nucleotidase. Many 5′-nucleotidases from snake venoms are homologous to the ecto-5′-nucleotidases, also known as CD73, which carry a GPI anchor that attaches proteins to membranes [83], correlating with an early description of membrane vesicles containing membrane ecto-5′-nucleotidases. In addition to disrupting the host’s immune system, it has also been proposed that SVEVs contribute hugely to the long-term effect or relapse for systemic envenoming [91].

#### 2.2.2. Spider Venom EVs

EVs have been discovered in the venom of spiders. The protein composition of venom proteins in spider venom and gland excretion is well known. A study by Xun et al. [92] identified the presence of EVs within the venom of the tarantula species *Ornithoctonus hainana* using transmission electron microscopy. They successfully isolated and purified the venom EVs (HN-EVs) through density gradient centrifugation, finding that they had a size range between 50 and 150 nanometers and expressed the arthropod EV marker protein, Tsp29Fb [92]. An LC-MS/MS analysis of HN-EVs revealed 150 proteins, which were divided into three groups according to their potential function: conservative vesicle transport-related proteins, virulence-related proteins, and other proteins of an unknown function. They found that HN-EVs have hyaluronidase activity and inhibit the proliferation of human umbilical vein endothelial cells (HUVECs) by affecting the cytoskeleton and cell cycle [92].

#### 2.2.3. Wasp Venom EVs

EVs from *Leptopilina endoparasitoid* venom contained proteins and peculiar vesicles called venosomes. These vesicles seem to have a unique extracellular biogenesis in the wasp venom apparatus, where they acquire specific secreted proteins/virulence factors and act as a transport system to deliver these compounds into host lamellocytes [93]. Similarly, *L. endoparasitoids* venom vesicles were found to transport and target virulence factors, dependent on the association with LbGAP and the flotillin-1 association, which functions in endocytosis. These phenotypes were not dependent on clathrin or micropinocytosis, suggesting that the presence of an early endosomal compartment was able to bypass Rab5 to impair the encapsulation of the parasitoid eggs by the lamellocytes of their *Drosophila melanogaster* host larva [94].

#### 2.2.4. Tick Venom EVs

Tick saliva contains numerous protein families commonly found in venomous taxa, and many genera of *Ixodida* can cause paralysis and other toxicoses; however, despite this, ticks are rarely regarded as venomous creatures [50]. Numerous families of tick salivary proteins and their previously identified roles are similar to those of proteins found in the venoms of snakes, bees, scorpions, platypuses, and spiders [50]. Several tick species, such as *Dermacentor andersoni*, *Argas walkerae*, and *Ixodes holocyclus*, were reported to have toxins in their saliva that cause paralysis. These species are known to induce paralysis by interfering with the synthesis of acetylcholine, thus reducing its release at the neuromuscular junction [51,52,53]. Interestingly, ticks were found to secrete EVs, such as exosomes, which facilitate the transfer of flavivirus RNA and proteins to host cells. Zhou et al. [55] discovered the existence of refined neuronal and arthropod exosomes, ranging in diameter from 30 to 200 nm. Exosomes produced from arthropod, mouse, and human cells were shown to contain both positive and negative strands of LGTV RNA and viral envelope proteins, suggesting that exosomes carry viral proteins. Exosomes derived from tick and mammalian cells included viral RNA and proteins that were safe, highly contagious, and replicative in every assessment [55]. This study uncovered the new roles of EVs to function in vector defense mechanisms against tick-borne virus infections and anti-viral pathways.

Furthermore, a recent study was the first to isolate, quantify, and characterize proteins in EVs from *Bubalus bubalis* infected with *Theileria* spp. [95], a parasitic infection spread by tick species [96]. De Pontes et al. [95] discovered buffalo sera EVs ranging in size from 124 to 140 nm and identified 306 proteins via LC/MS. This research enhances our understanding of the host–parasite relationship, advancing our awareness of the host immune response and the mechanisms by which *Theileria* evades detection. These findings provide new insights into potential EV candidate markers for improving the production of safe biological products derived from buffaloes.

## 3. Venom EV Pathogenesis: New Evidence

**Figure 2:**
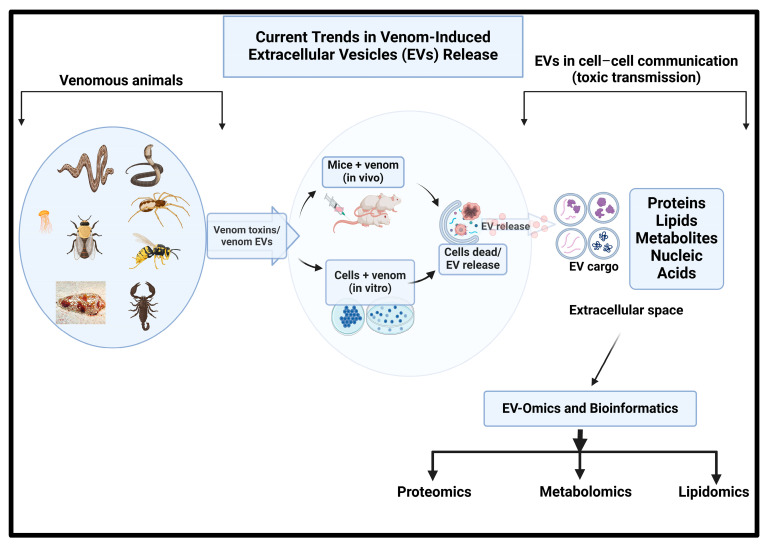
Model of EVs and exosomes in toxin transmission.

Recent findings have shown that the snake venom- or single toxin-induced EVs isolated from plasma and peritoneal exudate can contain unique proteomic profiles for inflammatory response, and single toxin-induced EVs from human neutrophils can result in proinflammatory mediator release (Figure 2). The proteomic analysis of exudate- and plasma-derived EVs using LC-MS/MS revealed the upregulation and downregulation of proteins involved in cell adhesion, cytoskeleton rearrangement, signal transduction, immune responses, and vesicle-mediated transports [88]. Interestingly, the miRNA cobra-derived SVEVs induced an envenomation-like phenotype, resulting in significant similarities to venom-induced toxicities [97]. Furthermore, in vitro studies have, for the first time, reported the *Loxosceles* venom’s ability to stimulate the production of EVs in various human cell lineages. Components of the *Loxosceles* venom were also detectable in the cargo of these vesicles, suggesting that they may be implicated in the process of extracellular venom release [98].

### Cellular EVs from Venom and Single Toxin Exposure

The mass spectrometry-based proteomics and bioinformatic analysis of EVs isolated from mice plasma 48 h after the injection of the venoms of *C. atrox* and *C. o. helleri* revealed upregulated responses associated with cytochrome P450, lipid metabolism, acute phase inflammation, immune responses, and heat shock responses. In contrast, downregulated proteins were associated with mitochondrial electron transport, NADH metabolism, the TCA cycle, the cortical cytoskeleton, endoplasmic reticulum stress, and oxidative reduction processes [4]. Altogether, this analysis provides direct evidence for the EV composition and its effects, highlighting the pathophysiological changes in an envenomated organism through its extracellular vesicles.

The study of the EV response to envenomation is an emerging area with little information on EVs’ participation and role during systemic envenoming. *Calloselasma rhodostoma* venom-derived LAAO (Cr-LAAO) was reported to affect the release of exosomes from human neutrophils, leading to the understanding of exosome activation patterns, ultimately revealing insights on the pathology of envenomation and potential therapeutic approaches [99]. The proteomic analysis of the exosomes identified proteins related to regulating immune responses and blood coagulation, suggesting more evidence for immune cell modulations [99]. Similarly, our group identified proteins in peritoneal exudate and plasma EVs isolated from BALB/c mice following a 30 min period post-injection of *C. s. scutulatus* venom and its purified CRiSP (Css-CRiSP). The proteomic analysis of exudate- and plasma-derived EVs found the significant upregulation or downregulation of proteins involved in cell adhesion, cytoskeleton rearrangement, signal transduction, immune responses, and vesicle-mediated transports. Our data imply that svCRiSPs play a role in the acute venom response and contribute to both local and systemic toxicity in snakebite envenoming (SBE) [88]. In another report, the venom of *Loxosceles intermedia* stimulated the production and the release of EVs in human embryonic kidney cells (HEK-293) and human monocyte THP-1 cells [98]. Components of the *L. intermedia* venom (phospholipases D) were also detectable in the cargo of these vesicles, suggesting that they may be implicated in the process of extracellular venom release. EVs from venom-treated cells exhibited PLD activity, induced in vitro hemolysis in human red blood cells, and altered the HEK cell membranes’ permeability [98].

## 4. Looking Forward: Exposomics/Therapeutics Systems Biology

Recently, new advances in enrichment strategies (ultracentrifugation, size exclusion, and affinity strategies) have attempted to comparatively analyze the protein cargo of different EV populations, often comparing small EVs (exosomes) with larger EVs. While several common proteins have been identified, numerous unique proteins have also been detected, primarily suggesting potential subtype markers and distinct and/or commonly associated molecular pathways [100]. To further the understanding of the pathophysiology of different disease conditions and their relationship to the environment, proteomics techniques have been used over the years in a variety of approaches to monitor or understand quantitative changes in protein expression that may occur due to diseases, the body’s response to drug or toxin exposure, and the impacts of these changes [101,102]. The advancement in quantitative and qualitative mass spectrometry-based proteomics has tremendously contributed to the understanding of the role of EVs, particularly in cancer and neurodegenerative disorders [103,104,105,106]. Recently, researchers have begun exploring the role of EVs in toxicology research through quantitative and qualitative mass spectrometry-based proteomics of exposomes from environmental and animal toxins [4,69,83,92,107].

Metabolomics approaches in biomedical sciences and system biology focus on the measurement of metabolomes/metabolites as chemical responses to changes in biological processes [108,109]. Metabolites are small molecules that are intermediates or end products of metabolic processes within organisms. Metabolites are involved in a variety of chemical reactions essential for cellular function, holding important biological information, making them one of the most predictive molecules of the phenotype [110]. They are critical for understanding physiology, disease mechanisms, and therapeutic developments. The advancement in analytical biology and bioinformatics, such as mass spectrometry, spectroscopy, and atomic absorption spectrometry, has presented the measurements of metabolomes in biology systems as an important aspect in understanding the chemical responses and metabolic pathways involved in the pathophysiology of diseases and the toxicological responses of the biological system, including envenomation [108,111,112]. Additionally, the measurement of the metabolite abundance can serve as an important diagnostic biomarker of disease and toxicity progression [109]. In toxicological research, the targeted and untargeted identification and monitoring of metabolites and metabolic processes are crucial for diagnosis and treatment, especially in forensic toxicology and medicine [113]. Metabolomics plays a key role in monitoring and assessing toxicity from environmental chemicals and drugs [114]. The ability of EVs to mediate cellular signaling by carrying nucleic acids, proteins, lipids, and cellular metabolites between cells makes them an important biological target in metabolomics. Additionally, metabolic tissues and organs can produce EVs, and the abundance and expression of EVs and their cargo have been reported to change in response to systemic metabolic alterations [115,116].

### 4.1. Novel Treatments for Snakebite Envenoming (SBE)

An estimated 2.5 million snakebite envenomings occur worldwide every year, resulting in the death or permanent disability of at least 500,000 people, although the true impact of envenomation is certainly underestimated [117,118]. The clinical manifestations of envenoming range from severe tissue damage (e.g., muscle necrosis, skin blistering, and inflammation), which can lead to permanent disability or limb amputation, to life-threatening systemic effects (including paralysis, hemorrhages, and permanent kidney damage) or death [119]. Most SBEs occur in Africa, Asia, and Latin America and disproportionately affect agricultural workers and children in low-income, rural communities [2,117]. For these reasons, the World Health Organization added snake envenoming to its priority list of neglected tropical diseases in 2017 [120]. For envenomated individuals, rapid access to high-quality, highly effective therapeutics can be the difference between life and death. Currently, the only antidotes to SBEs are antivenoms produced by hyper-immunizing large animals, such as horses, with one or more venom and then harvesting blood plasma, which contains antibodies against the venom targets. While lifesaving, existing antivenoms have several major limitations, including their poor efficacy, batch-to-batch variability, and high cost [121]. Even when antivenoms are available, their intravenous administration and the risk of severe adverse reactions restrict treatment to clinical environments, delaying or preventing treatment in areas with poor transport and communications infrastructure. A transformative change is necessary if we are to supply safe, affordable, and clinically effective antivenoms to the populations in the greatest need of them, enabling rapid deployment in the event of SBEs. Although many efforts are underway, including the repurposing of chelating compounds and small molecules and the use of human-based monoclonal antibodies, another novel approach could be in the form of targeting pathogenic EV biogenesis and release during systemic envenoming.

### 4.2. Effective Treatments for Snake Envenomation Are an Urgent Medical Need: EV Inhibitors

**Figure 3:**
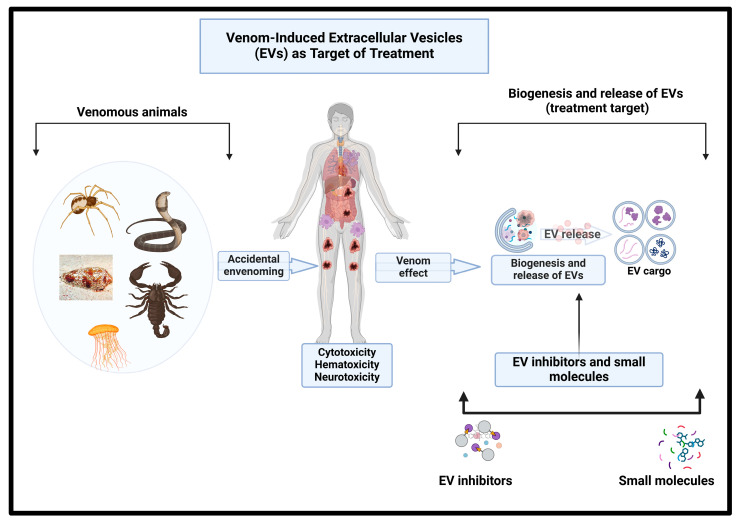
Proposed model of EVs as target of treatment.

The major toxins responsible for the systemic and lethal effects of venom are the SVMPs, SVSPs, svPLA_2_s, three-finger toxins (3FTxs), and LAAOs [122]. However, the most immediate and most acute local effects of snakebites are caused by the actions of non-enzymatic toxins found in the snake venom, such as CTLs and CRiSPs (Figure 3). Additionally, recent evidence suggests that SVEVs or venom-induced EVs contribute to the spread of envenoming [4,91]. These nanoparticles produced from the cells and the venom gland of snakes contain protein, RNA/miRNA, and metabolite cargoes that facilitate cell–cell communication (Figure 3). EV inhibitors have recently been broadly categorized into two groups based on their mechanism of action, those that affect the EV uptake (e.g., calpeptin) and those that affect EV biogenesis (e.g., imipramine) [123]. The involvement of EVs in disease development and progression has prompted many researchers to focus on their biogenesis and uptake inhibitors as potential therapeutics for various diseases. Currently, numerous biological agents are under investigation as EV inhibitors. Alone or combined with conventional chemotherapy, small-molecule EV inhibitors can disrupt EV biogenesis, release, and uptake in cancer and other diseases [124]. These attributes make them potential therapeutics for preventing the pathogenesis and/or progression of snake venom toxicities. Aside from having potential therapeutic advantages over conventional antivenoms, EV inhibitors also present the potential to serve as standard controls in the screening of other antivenom candidates (Figure 3).

## 5. Future Perspectives on EVs in Toxicology

We addressed the potential for identifying biomarkers from EVs specific to environmental exposure by comparing the types of exposure, frequency, cellular capacity, and new technological advances that have led to novel insights into various diseases. In addition, the molecular function of some of the described proteins suggests a central role for SVEVs in the cytotoxicity of snake venom, shedding new light on the envenomation process. We reviewed the progress and the importance of identifying and quantifying nucleic acids, proteins, and metabolites from the EVs released due to animal toxins, including drug toxicity. Significant advancements in physical, chemical, and biological analytical techniques have introduced progressive concepts in multi-omics technologies, including proteomics, metabolomics, genomics, and transcriptomics [125]. Proteomics and metabolomics are important omics techniques that utilize advancements in high-resolution mass spectrometry and the statistical power of bioinformatics to understand the expression of different proteins and metabolites in biological responses and metabolic processes, respectively, thereby presenting an opportunity to understand the pathophysiology of diseases and the exposomes of plant, animal, and environmental toxins. Thus, EVs’ DNA, RNA, protein, and metabolite contents have established a link between omics, EVs, and toxicology.

The study of toxicology using proteomics and metabolomics could be essential in understanding the biological systems’ responses to various environmental, plant, and animal toxins, making them important tools for diagnosing and the prognosis of toxicity, especially for emerging toxicants [126,127]. Additionally, assessing the body’s susceptibility to exposure and the characterization of altered biological and metabolic pathways could also provide insights into the potential links between toxicant exposure and other diseases. It is well established that an effective way to analyze (both quantitatively and qualitatively) the abundance of proteins and metabolites, including DNA and RNA, is by isolating and purifying EV cargo after toxicant exposure. The presence of EVs in plasma and their ability to downregulate or upregulate during exposure has further highlighted their importance in proteomics and metabolomics. Additionally, the advancements in mass spectrometry, in bioinformatics, and in artificial intelligence have provided opportunities to explore and analyze large data datasets, aiding in the diagnosis, treatment, and understanding of links between toxicant-induced toxicity and chronic diseases. The omics fields of toxicology, proteomics, and metabolomics offer new possibilities for analyzing EV cargo through multi-omics mass spectrometry and bioinformatics. These analyses provide valuable information on the role of EVs in the pathophysiology of diseases and the exposomes of toxicants by examining biological and metabolic responses expressed through the proteins, metabolites, and nucleic acids contained in EVs.

Alone or combined with conventional therapy, small-molecule EV inhibitors can disrupt EV biogenesis, release, and uptake in cancer and other diseases [124]. These attributes make them potential therapeutics for preventing the pathogenesis and/or progression of acute or chronic injury caused by environmental toxins and SBE. Additionally, EV inhibitors hold promise as potential candidates for screening new drug therapies.

### Limitations

The multi-omics-based analysis of extracellular vesicles is not without limitations. First, the variability of different EV enrichment techniques has previously presented a high percentage of certain types of EVs compared to others; this issue may affect reproducibility, especially with the introduction of new techniques of EV isolation [128]. Additionally, the storage of EVs has been a challenge; the EV content varies when stored in different conditions, which might also affect batch-to-batch variation [60,129].

Omics techniques have greatly advanced research in system biology, providing researchers with avenues to explore biological systems in a more holistic way using large-scale data across all biological levels, including DNA, RNA, proteins, and metabolites. However, there are still a lot of limitations, especially in the lack of generally standardized protocols for sample preparation, false positives, data complexity, and interpretations that require advanced bioinformatics techniques. Additionally, the absence of information for many experimental species presents a huge gap between the interpretation and integration of large data in preclinical to clinical research designs across different omics platforms. The field of venom/antivenom research has positively explored omics techniques, more specifically proteomics, metabolomics, and genomics. It is very important for researchers to understand the limitations of omics techniques and create appropriate and repeatable data analysis approaches, standardized procedures, and collaborative initiatives to combine, analyze, and visualize data from various omics platforms.

## 6. Conclusions

In summary, the exploration of EVs in venom biology opens new frontiers in our understanding of how venoms exert their potent biological effects. EVs serve as carriers of diverse molecular cargo—proteins, lipids, RNA, and other bioactive molecules—facilitating targeted delivery and enhancing venom efficacy. Investigating their role in immune modulation, tissue targeting, and interspecies communication offers exciting opportunities to develop novel therapeutic applications. Moreover, the intersection of venom research with EV biology may shed light on previously unexplored mechanisms of venom action and provide insights for drug delivery strategies. As research advances, this emerging field promises to bridge gaps in toxinology and extracellular vesicle science, revealing new layers of complexity in venom function and potential avenues for medical innovation.

## Figures and Tables

**Table 1:** Types and families of venomous species and the diversity and clinical effects of their toxins.

Venomous Species	Family	Pathophysiologically Active Toxins	Clinical Effects of Toxins	References
Snakes	Viperidae, Elapidae, Colubridae, Atractaspididae, Hydrophidae	Phospholipase A_2_ (PLA_2_), snake venom metalloproteinases (SVMPs), snake venom serine proteinases (SVSPs), three-finger toxins (3FTxs), C-type lectin (CTL), L-amino acid oxidase (LAAO), cysteine-rich secretory proteins (CRiSPs), ohanin, cathelcidin, snake venom waprin, serine proteinase inhibitors, and cobra venom factor (CVF)	Tissue necrosis and inflammation, anti- and pro-coagulant effect, hemolysis, skeletal muscle paralysis, hypotensive effect, hyperalgesia, neurotoxicity	[4,6,7,22]
Scorpions	Buthidae, Hydrophidae, Hadruridae, Belisariidae, Diplocentridae, Euscorpiidae, Luridae, Urodacidae, Vaejovidae, Scorpionidae	Phosphodiesterases, phospholipases, hyaluronidases, glycosaminoglycans, histamine, and serotonin	Hyperalgesia, inflammation, neuromuscular excitability, hypotensive effects	[10,24,26]
Spiders	Theridiidae, Sicariidae, Phoneutria, Scytodidae, Latrodectidae, Actinopodidae	Neurotransmitter modulators, immune modulators, ion channel blockers, and cysteine-rich peptides	Edema, blood vessel permeability, neuromuscular excitability, intravascular hemolysis, and dermonecrosis	[9,32,33,34,36]
Bees/wasps	Apidae, Vespidae, Pelecinidae	Bees: melittin, apitoxin, PLA_2_, histamine, hyaluronidase, and serotonin phospholipids, sugars, and biogenic amines Wasps: PLA_2_, antigen 5, mastoparan, eumenitin, eumenitin-R, rumenitin-F, hyaluronidase, α-glucosidase, anoplin, and decoralin	Hyperalgesia, hemolysis, neurotoxicity, neuromuscular effect	[41,43,44]
Cone snails	Conidae	Neurotransmitter modulators and ion channel modulators (nAChR conotoxins, Na^+^ conotoxins, K^+^ conotoxins, Ca^2+^ conotoxins), conantokins, chiconotoxin, and conopressins	Neuromuscular paralysis, neurotransmitter excitability, pain signal blockage	[15,37,38,39,40]
Venomous fishes	Scorpaenidae, Characidae, Synancejidae, Mobulidae	Hyaluronidase, pain-producing factor, orpotrin, porflan, cardioleputin, trachynilysin, stonustoxin, verrucotoxin, and neoverrucotoxin, vasoactive kinin, 5-hydroxytryptamine, histamine, and catecholamines	Hemolysis, muscular paralysis, hyperalgesia, dermonecrosis, cardiovascular damage	[16,17,49]
Ticks	Argasidae, Ixodidae	Lectins, cystatins, lipocalins, hyaluronidase, PLA_2_, kunitz-like peptides, and metalloproteases	Inhibiting hemostasis, modulation of host immune system, pain signaling blockage	[50,51,54,55]

## Data Availability

No new data were created or analyzed in this study.
